# Hematoma cavity separation and neomembrane thickness are potential triggers of recurrence of chronic subdural hematoma

**DOI:** 10.1186/s12893-022-01687-9

**Published:** 2022-06-20

**Authors:** Hongbin Liu, Rudan Yan, Fei Xie, Seidu A. Richard

**Affiliations:** 1 Department of Neurosurgery, The First People’s Hospital of Ziyang, No. 66, Rende West Road, Ziyang, Sichuan 641300 People’s Republic of China; 2 Department of Otolaryngology, Head and Neck Surgery, The First People’s Hospital of Ziyang, No. 66, Rende West Road, Ziyang, 641300 Sichuan People’s Republic of China; 3 Department of Medicine, Princefield University, P. O. Box MA 128, Ho-Volta Region, Womenu, Ghana

**Keywords:** CSDH, Craniotomy, Craniostomy, Connector, Endoscopy

## Abstract

**Background:**

Chronic subdural hematoma (CSDH) is the anomalous and encapsulated accumulation of fluid of complex origin consisting of old blood, mostly or totally liquified and cerebrospinal fluid (CSF) in the subdural space usually after a head injury in the elderly. Almost all the research on surgical techniques and endoscopic assisted evacuation of CSDH focused on the just the evacuation and not abnormal anatomical structures that causes recurrences.

**Objectives:**

We investigated abnormal anatomical structures that triggers recurrence of CSDH during craniotomy as well as burr-hole craniostomy with endoscopic assistance.

**Materials and methods:**

We retrospectively analyzed all patients with CSDH who underwent craniostomy and burr-hole craniotomy with endoscopic assisted evacuation of hematoma between April 2017 and November 2020 at our institution. Clinical data obtained was categorized into patient-related, radiology as well as surgery and endoscopic evaluations.

**Results:**

A total of 143 patients (109 men and 34 women) aged 43–94 years (mean age, 68.35 years) with CSDH were included in this study. We observed a recurrence rate of 4.9% (7/143). Recurrences occurred between 2 and 6 months after the operation in patients with recurrences. Our data revealed that, age, hypertension, history of injury, diabetes, antiplatelet or anticoagulant use were not associated with hematoma recurrence. Nevertheless, all the patients with recurrence of hematoma were males. Interestingly, our univariate and multivariate analyses found neomembrane thickness and hematoma cavity separation as independent risk factors (OR,45.822; 95% CI,2.666-787.711; p = 0.008) for the recurrence of CSDH (p < 0.05). Also, we observed thickened membranes connecting/separating the dura and the thickened arachnoid/pia matters in all the 7 patients with hematoma recurrence.

**Conclusions:**

The treatment of patients with CSDH ought to include the identification and resection of abnormal thickened membranes connecting/separating the dura and the thickened arachnoid/pia matters to avoid recurrence. Comparatively, endoscopy showed hematoma cavity separation or neomembrane thickness just as seen during craniotomy.

## Introduction

Chronic subdural hematoma (CSDH) is the anomalous and encapsulated accumulation of fluid of complex origin consisting of old blood, mostly or totally liquified and cerebrospinal fluid (CSF) in the subdural space usually after a head injury in the elderly [1–5]. Most often, the accumulated fluid exerts pressure on the brain tissues resulting in neurologic complications [2, 3]. Initially, CSDH was perceived as a gradual as well as recurring hemorrhage caused by ruptured cortical bridging veins during the trauma [4, 6, 7]. Currently, multiple factors such as inflammation, angiogenesis, recurrent micro-hemorrhages, as well as local coagulopathy in the subdural space has been implicated as the cause of the CSDH [6, 8, 9].

Noteworthily, inflammatory reactions often trigger the formation of hematoma, re-bleeding as well as maintenance and liquification of the hematoma [6, 8, 9]. This process often results in the formation of a primary neomembrane with rich vasculature [1, 10]. The neomembrane typically consists of a thick outer neomembrane as well as a thin neomembrane surrounding the blood which also successively liquidifies [1, 10]. The neomembrane, restrict the brain tissue from expanding leading to subdural hematoma cavity that persists and triggers recurrence of the CSDH [1, 10].

Clinically, the patients often present with symptoms such as headaches, behavioral changes, focal deficits, seizures as well as hemiparesis [11, 12]. Non-contrast Computed tomographic (CT) scan is usually the gold-standard radiology modality use in detecting this anomalous liquified blood in subdural space [6]. Conservative treatment with ACE-inhibitors, corticosteroids, atorvastatin as well as tranexamic acid have also been used in the management of CSDH [1, 13–15]. Nevertheless, surgical treatment is the most preferred treatment option of CSDH particularly in cases that present with mass effect [1].

Craniotomy, burr-hole craniostomy as well as twist-drill craniostomy, are the well-known surgical techniques for the treatment of CSDH [1, 16–18]. Nevertheless, about 2.5–33% of recurrence rates are associated with the treatment modalities above [6, 19, 20]. Endoscope-assisted evacuation of CSDHs is an accepted technique, though not generally used [21–28]. It is an amalgamation of burr-hole evacuation of CSDH as well as inspection of the subdural space with an endoscope [16].

The above technique is advantageous because, the placement of the catheter is often under visual control, detection of septations as well as timely recognition of essential cortexes or vessel injury during surgery [16]. Almost all the research on surgical techniques and endoscopic assisted evacuation of CSDH focused on the just the evacuation and not abnormal anatomical structures that causes recurrences. Thus, our study explores factors that triggers recurrence of CSDH during and after treatment.

## Materials and methods

### Patients

We retrospectively analyzed all patients with CSDH who underwent craniotomy and burr-hole craniostomy with endoscopic assisted evacuation of hematoma at the Department of Neurosurgery, Zi Yang first people’s hospital, China, between April 2017 and November 2020. This study was reviewed and approved by our Institutional Research Review Board. The patients as well as their relatives were dually informed about our intention to involve them in a study and they fully concerted to the use of their information. All the patients and their relatives signed written informed consents. Factors triggering recurrence of CSDH were divided into three broad categories such as patient comorbid factors, hematoma subtypes on radiology as well as abnormal anatomical structures. Clinical data obtained was categorized into patient-related, radiology as well as surgery and endoscopic evaluation. Patient-related data such as age, sex, history of injury, hypertension, diabetes, antiplatelet or anticoagulant use were obtained and documented at the time of admission in the ward. All the patients were referred to our hospital from periphery hospitals with history of CSDH.

### Radiological evaluation

The diagnosis of CSDH patients was re-established by CT scan on admission. Radiology related data such as unilateral or bilateral hematoma, hematoma thickness, density of the hematoma, midline brain shift and brain atrophy were obtained and documented. Also, initial detection of hematoma cavity separations and neomembrane thicknesses were established on CT scan. Neomembrane thicknesses were seen as thickened dura structures while hematoma cavity separations were seen as two or more subdural cavities. All hematomas were classified into four types according to their density on CT scan: Low-density (< 25 Hounsfield unit [HU]), iso-density (25–35 HU), hyper-density (> 35 HU), and mixed-density [29]. Brain atrophy was classified into the three types based on CT findings: no or mild atrophy, moderate atrophy, such as dilated sulci, and severe atrophy, such as widely dilated sulci and subdural space [30]. Additionally, postoperative CT scans were performed within 3 days after surgery.

### Surgical management

All patients who underwent surgery were identified as CSDH before surgery. Patients either underwent a single burr-hole craniostomy with endoscopic assisted hematoma evaluation or craniotomy under general anesthesia and closed-system drainage based on liquefication of the hematoma on preoperative CT scan. Bleeding bridging veins are more likely observed in liquefied CSDH necessitating occlusion during craniotomy compared to none liquefied CSDH. All patients were placed in lateral position during the operation and after removing the bone flap, the hematoma was flushed out via irrigation with warm physiological saline solution to reduce postoperative gas accumulation and residual of the hematoma. Neomembrane thicknesses, hematoma cavity separations as well as all other radiological parameters were reestablished intraoperatively. Intraoperation, neomembrane thicknesses were seen as thickened dura structures relative to the normal dura while hematoma cavity separations were seen as two or more subdural cavities. Also, patients were observed under flexible endoscopy for the presence of a hematoma cavity and the formation as well as neomembrane thickness in the single burr-hole craniostomy treated patients. In all patients, drainage catheters were removed 48 h whether or not residual hematomas were seen on postoperative CT scans after the operation.

### Followed-up and evaluation of recurrence

All patients were followed monthly for 6 months after surgery at outpatient to establish recurrence of CSDH. CT scan were performed in all the patient’s whether they present with neurological symptoms or not to establish recurrence during follow-ups. Recurrence was defined as any increase in the volume of ipsilateral subdural hematoma on CT scan after removal of drainage system whether the patient was symptomatic or asymptomatic. Conservative treatment was not adapted in our cases with recurrence because this treatment modality is not part of our routine treatment of CSDH. After the first 6 months, all the patients were subsequently followed for at least 2 years. We had no missing data and no patients were lost on follow-ups.

### Statistical analysis

We used commercially available software (IBM SPSS Statistics for Windows, Version 25.0; IBM Corp, Armonk, New York, USA) for statistical analyses. Chi-square test or Fisher’s exact test was used for univariate analyses. In logistic regression analysis, adjustments were made for age, gender, diabetes, hypertension, antiplatelet and/or anticoagulant use, history of trauma, bilateral hematoma, brain atrophy, hematoma thickness and midline shifting. Model covariates were determined by descriptive statistics and previous studies. The relationship of each predictive factor and CSDH recurrence was presented as odds ratio (OR) and 95% confidence interval (CI). A p-value < 0.05 was defined as statistically significant.

## Results

### Patients demographic characteristics

A total of 143 patients (109 men and 34 women) aged 43–94 years (mean age, 68.35 years) with CSDH were included in this study. The baseline characteristics are described in Table 1. Among them, 76 patients admitted haven experienced one or more episodes of apparent head trauma while 67 could not remember traumatic events. The time interval between trauma and the first operation was 10–90 days (mean 24 days). Six patients where on aspirin, seven patients were on clopidogrel and four patients were on warfarin. Our data revealed that, age, hypertension, history of injury, diabetes, antiplatelet or anticoagulant use were not associated with hematoma recurrence **(**Tables 1, 2**)**. Nevertheless, all the patients with recurrence of hematoma were males.

**Table 1:** Summary of baseline characteristics in no recurrence group and recurrence group in 143 patients with CSDH

Factor	No recurrence (%)	Recurrence (%)	p value
Total	136 (95.1%)	7 (4.9%)	
Sex			0.231
Female	34 (23.8%)	0 (0.0%)	
Male	102 (71.2%)	7 (4.9%)	
Age (years)	70.75 ± 8.91	64.14 ± 15.52	0.418
Diabetes	22 (15.4%)	2 (1.4%)	0.736
Hypertension	60 (41.9%)	1 (0.7%)	0.244
Antiplatelet and/or anticoagulant use	16 (11.2%)	1 (0.7%)	0.436
Bilateral hematoma	16 (11.2%)	0 (0.0%)	1.000
History of trauma	73 (51.0%)	3 (2.1%)	0.949
Brain atrophy			0.758
Mild	102 (71.3%)	5 (3.5%)	
Moderate	26 (18.2%)	2 (1.4%)	
Severe	8 (5.6%)	0 (0%)	
Hematoma thickness (cm)	1.97 ± 0.56	2.13 ± 0.52	0.482
Midline shift (cm)	0.86 ± 0.55	0.69 ± 0.51	0.426
Density of hematoma on CT			≺ 0.001
Isodensity hematoma	64 (44.8%)	0 (0%)	
Low-density hematoma	26 (18.2%)	0 (0%)	
High-density hematoma	23 (16.1%)	1 (0.7%)	
Mixed density hematoma	23 (16.1%)	6 (4.2%)	
Neomembrane in CT	13 (9.1%)	4 (2.8%)	0.003
Hematoma cavity separation in CT	11 (7.7%)	4 (2.8%)	0.002
Hematoma cavity separation or Neomembrane in endoscopy	34 (23.8%)	6 (4.2%)	0.001

*CSDH* chronic subdural hematoma, *CT* computed tomography, % percentage, *CM* centimeter

**Table 2:** Logistic regression analysis of predictive factors for recurrence of CSDH

	p value	OR (95% CI)
Model 1		
Gender	0.998	NA
Age (years)	0.362	0.949 (0.847–1.063)
Hypertension	0.085	0.060 (0.002–1.474)
Diabetes	0.514	0.419 (0.031–5.718)
Antiplatelet and/or anticoagulant use	0.101	14.836 (0.590-372.953)
Bilateral hematoma	0.998	NA
History of trauma	0.861	0.839 (0.117–6.008)
Mild brain atrophy	0.320	1
Moderate brain atrophy	0.131	10.816 (0.491-238.388)
Severe brain atrophy	0.999	NA
Hematoma thickness	0.313	2.885 (0.368–22.592)
Midline shift	0.809	0.748 (0.071–7.850)
Neomembrane in CT*	0.019	56.135 (1.916-1644.653)
Model 2		
Gender	0.998	NA
Age (years)	0.551	0.963 (0.851–1.090)
Hypertension	0.162	0.109 (0.005–2.441)
Diabetes	0.572	0.488 (0.040–5.905)
Antiplatelet and/or anticoagulant use	0.099	14.848 (0.603-347.929)
History of trauma	0.891	0.872 (0.125–6.088)
Bilateral hematoma	0.998	NA
Mild brain atrophy	0.528	1
Moderate brain atrophy	0.259	6.586 (0.250-173.249)
Severe brain atrophy	0.999	NA
Hematoma thickness	0.347	2.715 (0.339–21.774)
Midline shift	0.586	0.498 (0.040–6.125)
Hematoma cavity separation in CT*	0.019	40.173 (1.833-880.678)
Model 3		
Gender	0.997	NA
Age (years)	0.266	0.914 (0.780–1.071)
Hypertension	0.161	0.114 (0.005–2.373)
Diabetes	0.797	1.409 (0.104–19.141)
Antiplatelet and/or anticoagulant use	0.869	1.285 (0.065–25.561)
Bilateral hematoma	0.998	NA
History of trauma	0.451	2.376 (0.251–22.522)
Mild brain atrophy	0.794	1
Moderate brain atrophy	0.498	3.968 (0.074-212.904)
Severe brain atrophy	0.999	NA
Hematoma thickness	0.704	1.593 (0.144–17.632)
Midline shift	0.446	0.287 (0.012–7.131)
Hematoma cavity separation or* Neomembrane during endoscopy	0.008	45.822 (2.666-787.711)

Results were derived from logistic models. Model 1, Model 2 and Model 3 were adjusted for age, gender, diabetes, hypertension, antiplatelet and/or anticoagulant use, bilateral hematoma, brain atrophy, hematoma thickness and midline shift, history of trauma

*OR* odds ratio, *CI* confidence interval, *NA* not available, *CSDH* chronic subdural hematoma, *CT* computed tomography

*Significant difference

### Radiological outcomes

**Fig. 1:**
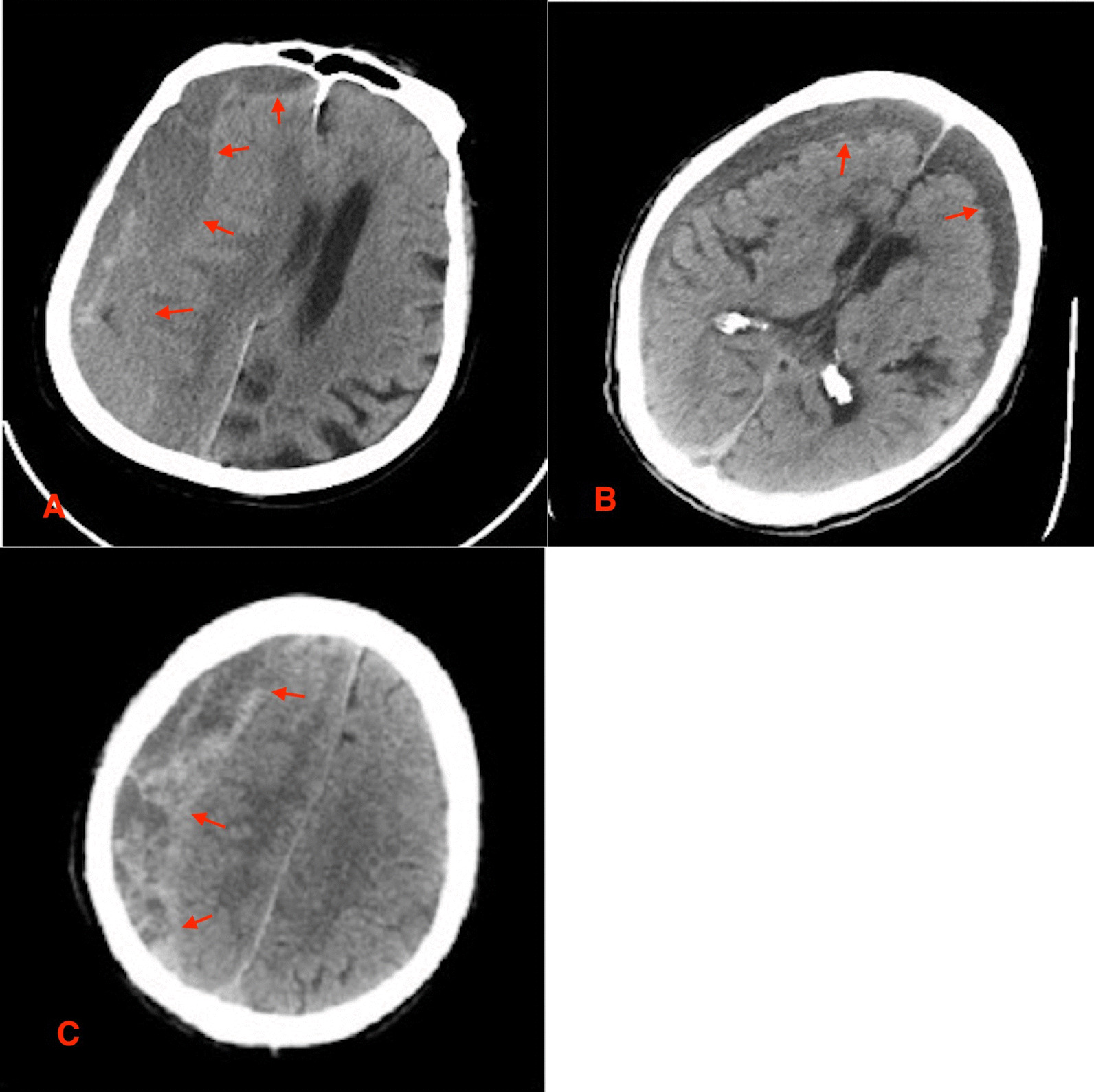
CT characteristic of patients: Hematoma cavity separation, new neomembrane and mixed density hematoma in CT. **A, B** hematoma cavity separation. **C** Mixed density hematoma. ***Red arrow***  boundaries of the abnormality

CT scan at admission showed that, the CSDH was left-sided in 75 patients, right-sided in 52 patients, and bilateral in 16 patients. We observed mixed density hematoma in 29 patients, iso-density hematoma in 64, high-density hematoma in 24, low-density hematoma in 26. CSDH was observed in 107 patients with mild brain atrophy, 28 patients with moderate brain atrophy and 8 patients with severe brain atrophy. We observed midline shifts in 123 patients. While 20 patients had no midline shifts on CT scan. Midline shift ranged from 0 to 2.2 cm (mean 0.85 cm). Hematoma thickness ranged from 8 to 33 cm (mean 1.98 cm). Hematoma cavity separation was found in 15 patients, and neomembrane thicknesses were detected in 17 patients. Out of 17 patients with neomembrane thickness, mixed density hematoma was observed in 11 patients, iso-density hematoma in 2, high-density hematoma in 1 and low-density hematoma in 3. Also, all the 15 patients with hematoma cavity separation showed mixed density hematoma. CT characteristic of patients are as shown in **(**Fig. 1A–C**)**. Also, characteristic or radiological outcomes is as shown in Table 1. Furthermore, unilateral or bilateral hematoma, hematoma thickness as well as midline shift were not associate with hematoma recurrence.

### Surgical and endoscopic outcomes

**Fig. 2:**
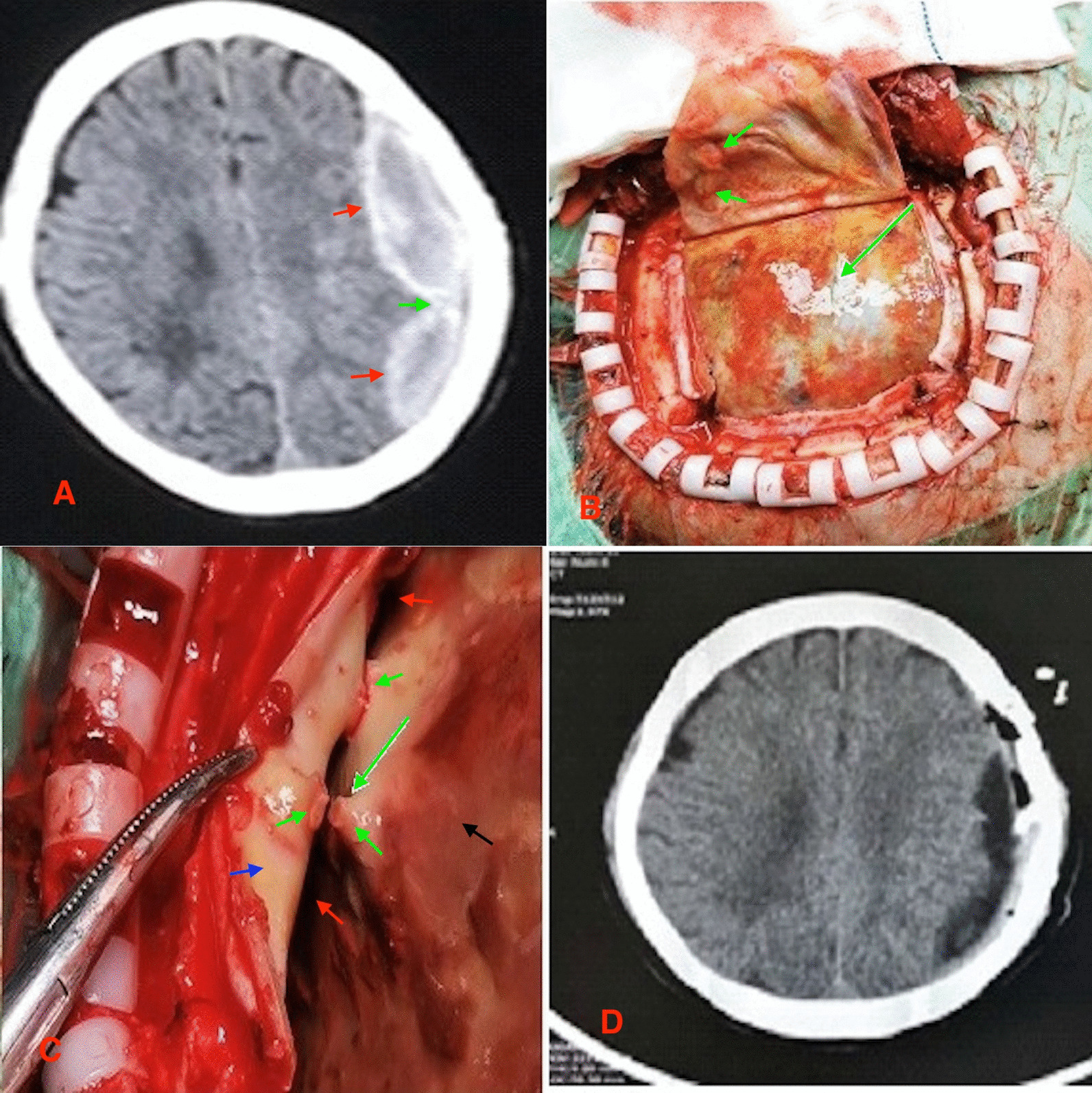
Preoperative CT scan, intraoperative images and postoperative CT scan on one of the patients without completely liquefied hematoma and hematoma cavity separation who underwent craniotomy. **A** preoperative CT image: ***Red arrow*** = None liquefied hematoma; ***Green arrow*** = thickened membranes connecting/separating the dura and the thickened arachnoid/pia matters. **B**, **C** Intraoperative images showing neomembrane, hematoma cavity separation as well as thickened membranes connecting/separating the dura and the thickened arachnoid/pia matters. **B**: ***Green*** arrows = thickened dura separators. **C**: ***Green arrows*** = thickened membrane connecting/separating the dura and the thickened arachnoid/pia matters, ***Red arrows*** = hematoma cavity separation, ***Black arrow*** = neomembrane and ***Blue arrow*** = dura. **D** Postoperative CT scan showing evaluation of the hematoma and separation of the thickened membranes connecting/separating the dura and the thickened arachnoid/pia matters

Total of 133 patients underwent single burr-hole craniostomy with endoscopic assisted hematoma evacuations and closed drainage while 10 patients underwent craniotomy (Fig. 2A). We resected the thickened neomembrane in two patients after craniotomy (Fig. 2B, C) and none of them experienced recurrence of the hematoma. Intraoperatively, we observed thickened membrane connecting/separating the dura and the thickened cortical surface comprising of arachnoid/pia matters (Fig. 2B, C) in the two patients above which were resected. Postoperative CT scan showed total evaluation of the hematomas **(**Fig. 2D**)**. None of patients developed surgical site complications after burr hole drainage.

**Fig. 3:**
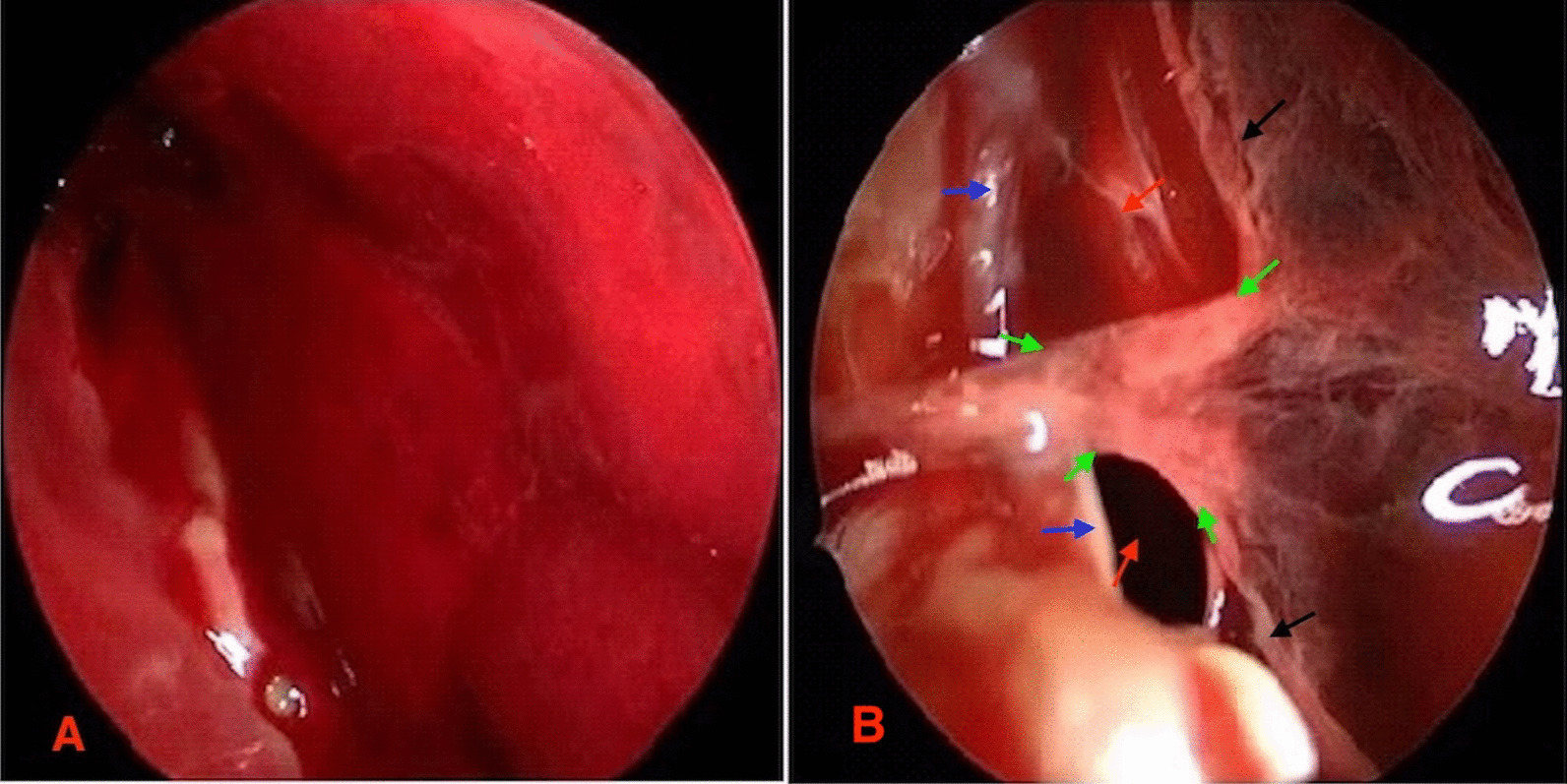
Intraoperative images showing the neomembrane, hematoma cavity separation in endoscopy as well as thickened membranes connecting/separating the dura and the thickened arachnoid/pia matters. **A** Endoscopic image showing the neomembrane. **B** Endoscopic image showing neomembrane, hematoma cavity separation as well as thickened membranes connecting/separating the dura and the thickened arachnoid/pia matters. ***Black arrows*** = neomembrane, ***Red arrows*** = hematoma cavity separation, ***Blue arrows*** = dura and ***Green arrows*** = thickened membranes connecting/separating the dura and the thickened arachnoid/pia matters

Hematoma cavity as well as the formation of neomembranes and their thicknesses were visible on endoscopy in the single burr-hole craniostomy treated patients **(**Fig. 3A, B). Quantitively, endoscopy showed hematoma cavity separation or neomembrane thickness in 40 patients. Comparatively, endoscopy showed hematoma cavity separation or neomembrane thickness just as seen during craniotomy (Figs. 2B, C, 3A, B). Similarly, on endoscopy, we observed the same thickened membranes connecting/separating the dura and the arachnoid/pie matter **(**Fig. 3B). These thickened membranes connecting/separating the dura and the thickened arachnoid/pia matters were often resected during endoscopy.

### Hematoma recurrence and patients’ outcomes after surgery

We did not observe recurrence within the first 3 days after operation during the hospitalization in all patients. We observed recurrence between 2 and 6 months after the operation in patients with recurrences. Overall, we observed recurrence of hematoma in 7 patients. Thus, the recurrence rate was 4.9% (7 of 143). Out of the 40 patients in whom endoscopy showed hematoma cavity separation or neomembrane thickness above, hematoma recurred in 6 of them.

One patient out of the 10 patients who underwent craniotomy had recurrence of the hematoma. Out of the 7 patients with hematoma recurrence, we observed mixed density hematoma in 6 patients during CT evaluation while one patient had high-density hematoma during CT scan evaluation **(**Table 1**)**. Also, in all the 7 patients with hematoma recurrence, we observed neomembrane thickness as well as hematoma cavity separation on CT scan. Furthermore, we observed thickened membranes connecting/separating the dura and the thickened arachnoid/pia matters in all the 7 patients with hematoma recurrence (Figs. 2C, 3B). We postulate that, inadequate or non-separation of thickened membranes connecting/separating the dura and the thickened arachnoid/pia matters could be a cause of the recurrence of hematoma. Thus, during the treatment of patients with CSDH, these membranes ought to be identified and resected.

Univariate and multivariate analyses found neomembrane thickness and hematoma cavity separation as independent risk factors (OR,45.822; 95% CI,2.666-787.711; p = 0.008) for the recurrence of CSDH (p < 0.05) **(**Tables 1, 2**)**. Two patients died after the first six-months follow-ups. One died of pneumonia while the cause of the death of the other was unknown. Thus, the mortality rate was 1.39% (2 of 143). No patient was lost on follow-ups. Patients with recurrence were re-treated via burr-hole craniostomy and endoscopic assisted hematoma evacuation with closed-system drainage with more focus on the resection of thickened membranes connecting/separating the dura and the thickened arachnoid/pia matters. We did not observe further or second recurrence during 2 years follow-ups.

## Discussion

CSDH is one of the most common post traumatic disorder managed by neurosurgeons [3, 31]. It is estimated that, about 8.2–14.1 per 100,000 persons often develop this disease per years in the general population [16, 32, 33]. CSDH is typically associated with good recovery after treatment although about 2.5–33% of patients present with recurrence necessitating re-operation [6, 31]. Also, repeated recurrences as well as refractory CSDH although rare, have been reported [31]. Thus, our study explores factors that triggers recurrence of CSDH during and after treatment. Factors triggering recurrence of CSDH were divided into three broad categories such as patient comorbid factors, hematoma subtypes on radiology as well as abnormal anatomical structures. We observed a recurrence rate of 4.9%. Recurrences occurred between 2 and 6 months after the operation in patients with recurrences.

Patient comorbid factors includes, advanced age, diabetes, hypertension, intracranial hypotension, alcohol consumption as well as antiplatelet or anticoagulant usage [1, 16, 32, 33]. Also, radiological risk factors associated with the recurrence includes, uni- or bilateral hematoma, preoperative hematoma thickness and midline shift, hematoma density and internal architecture, cerebral atrophy, as well as hematoma volume [6, 34–37]. Furthermore, procedural risk factors such as surgical technique, hemorrhage, postoperative posture, postoperative subdural accumulation of gas has also been implicated as causes of recurrence of CSDH [6, 16, 32, 33]. Interestedly, our univariate and multivariate analyses revealed that, age, hypertension, history of injury, diabetes, antiplatelet or anticoagulant use, were not associated with hematoma recurrence. Nevertheless, all the patients with recurrence of hematoma were males.

CT scan is the gold standard diagnostic modality for evaluating patients with CSDH. The evaluation of CT predictors is of great extra significance subsequent to other recognized clinical predictors [6, 35, 37]. In our study, CT scan was capable of detecting of hematoma cavity separations and neomembrane thicknesses. Neomembrane thicknesses were seen as abnormal structures relative to the normal dura while hematoma cavity separations were seen as two or more subdural cavities. Miah et al. with a meta-analysis observed that, hyperdense as well as mixed density hematoma were more linked to CSDH recurrence, as were laminar as well as separated architecture hematomas[6]. Furthermore, they indicated that, CSDH with high degree of hematoma thickness as well as midlines shift had more changes of recurrence [6]. They also found homogeneous hyperdense as well as mixed density hematoma to be more linked to recurrence rates [6].

Brain atrophy was observed in all the patients with CSDH. Most of the patients who developed CSDH had mild brain atrophy (107), 28 patients had moderate brain atrophy while 8 patients had severe brain atrophy. Recurrence occurred in 5 patients with mild brain atrophy and two patients with moderate brain atrophy. In our study, midline shift ranged from 0 to 2.2 cm (mean 0.85 cm). Nevertheless, midline shift was not associated with hematoma recurrence according to our analysis. Also, unilateral or bilateral hematoma in our CSDH patients were not associate with recurrence of the hematoma. Moreover, hematoma thickness ranged from 8 to 33 cm (mean 1.98 cm). Nevertheless, hematoma thickness was not also associated with recurrence of CSDH according to our analysis.

Iso-dense hematoma was the most prominent hematoma subtype according to our analysis followed by low-density hematoma and mixed density hematoma as well as high-density hematomas. Six patients with mix density hematoma had recurrence of the hematoma after treatment while one patient with high-density hematomas had a recurrence of the hematoma after treatment. We did not observe hematoma recurrence in patients with iso-dense hematomas and low-density hematomas.

Noteworthily, interplay of inflammatory cytokines is the widely accepted phenomenon causing recurrence of CSDH [6, 38–41]. Traumatic rupture of the arachnoid membrane often results in mild hemorrhage from bridging veins as well as brain contusion [1, 10, 42]. Subsequently, CSF gradually leaks into the collected blood via torn arachnoid membranes [1, 10]. The accumulated mix fluid triggers an inflammatory reaction in the subdural space[6, 38, 39]. This inflammatory process involves the release of cytokines as well as several other inflammatory markers which maintains the hematoma as well as re-bleeding tendencies [6, 39–41]. This process leads to the formation of a primary neomembrane with rich vasculature.

The neomembrane usually consists of a thick outer neomembrane as well as a thin neomembrane surrounding the blood which also subsequently liquidifies [1, 10]. The neomembrane, restrict the brain tissue from expanding resulting in subdural hematoma cavity that persists resulting in recurrence of the CSDH [1, 10]. We observed thickened membranes connecting/separating the dura and the thickened arachnoid/pia matters which sustains the separation of the hematoma cavities leading to recurrences of the hematomas after treatments.

Craniotomy, burr-hole craniostomy as well as twist-drill craniostomy are the widely used.

surgical treatment modalities for CSDH [16–18]. Currently, craniotomy under general anesthesia is mostly performed in organized hematomas or multilobulated as well as for recurrent incidents [16, 43]. Craniotomy is advantageous because, with a better exposure the surgeon is able to resect the outer capsule as well as neomembranes and also secure precise hemostasis [16, 43]. Although this surgical technique has the highest morbidity rate because of its invasiveness, it has the lowest recurrence rate compare to the other surgical modalities [16, 18, 43].

Our decision to perform craniotomy under general anesthesia and closed-system drainage was based on liquefication of the hematoma on preoperative CT scan. In our study, ten patients without completely liquefied hematoma and hematoma cavity separation underwent craniotomy. Intraoperatively, we observed a thickened membrane connecting/separating the dura and the thickened cortical surface comprising of arachnoid/pia matters in the two patients which we resected. Twist-drill craniostomy is mostly carried out at bedside under local anesthesia and amongst the three surgical techniques, it is the least invasive technique [16, 43]. Nevertheless, with this technique, profuse irrigation with saline, which appears to be advantageous in relation to the recurrence rate is often not achievable [16, 43, 44]. Burr-hole craniostomy is performed under either general or local anesthesia and it is usually the first-line treatment modality  [16, 17]. Nakaguchi et al. observed that frontal insertion of a draining catheter tip decreases recurrence of the hematoma [24].

Chihara et al. observed that, with the hematoma subtype, organized or septated CSDH frequently trigger recurrence of CSDH [45]. In cases of septated CSDH, a second burr-hole with robust as well as adequate irrigation often gives a good outcome in the second operation [31, 45]. Internal architecture types have also used for the categorization of CSDHs [6]. Nakaguchi et al. classified CSDHs into homogeneous, laminar, separated as well as trabecular, based on anticipated phases in the natural history of a CSDH [46]. Miah et al. found a higher recurrence risk in laminar as well as separated hematoma compared to others hematomas [6].

We observed hematoma cavity separation in 15 patients, and neomembrane thickness in 17 patients. Our univariate and multivariate analyses revealed that, neomembrane thickness and hematoma cavity separation were independent risk factors for recurrence of CSDH. Although organized or septated and separation of membranes in CSDH have reported as trigger of recurrence [6, 23, 31, 45, 46], no study reported a thickened membrane connector/separator between the dura and the thickened arachnoid/pia matters as cause of recurrence of the CSDH. We observed thickened membranes connecting/separating the dura and the thickened arachnoid/pia matters in all the 7 patients with hematoma recurrence.

Successful evacuation of subdural haematomas, pus as well as hygromas have been achieved with the use of endoscopic [21, 47, 48]. The endoscope assists in the assessment of the subdural space to ensure satisfactory clot evacuation as well as haemostasis, and to incise abnormal membranes as well as trabecula in instances of loculated CSDHs [21, 28, 47, 48]. Furthermore, during the procedure, the surgeon is able to irrigate the hematoma cavity, insert a drainage tube toward the frontal convexity to evacuate subdural air, avoid cortical laceration that often happen as result of blind manipulation, coagulate the source of bleeding as well as separate the inner membrane carefully to aid in brain expansion [22–26]. Nevertheless, during the procedure, the endoscope may cause injury to the brain cortex [16].

We performed single burr-hole craniostomy with endoscopic assisted hematoma evaluation and closed-system drainage in 40 patients. Hematoma cavity as well as the formation of neomembranes and their thicknesses were visible on endoscopy in the single burr-hole craniostomy treated patients. Out of the 40 patients in whom endoscopy showed hematoma cavity separation or neomembrane thickness, hematoma recurred in 6 of them. We observed thickened membranes connecting/separating the dura and the thickened arachnoid/pia matters in the 6 patients with hematoma recurrence on endoscopy. These thickened membranes connecting/separating the dura and the thickened arachnoid/pia matters were often resected during endoscopy. Comparatively, endoscopy showed hematoma cavity separation or neomembrane thickness just as seen during craniotomy. Thus, we postulate that, inadequate or non-separation of thickened membranes connecting/separating the dura and the thickened arachnoid/pia matters could be a cause of the recurrence of hematoma.

Mobbs et al. suggested that, in certain peculiar cases, the endoscopy could be used to assist in recognizing neomembranes intra-operatively, as well as perhaps aid in its treatment either via endoscopy-guided obliteration of the neomembrane or conversion to a normal craniotomy [23].

Chen et al. indicated that, pre-operative CT scans showing inner subdural membranes may guide one to target the treatment to allow for the release of this inner subdural membranes and that, mini craniotomy with careful fenestration of the inner membrane is very effective treatment [49]. Their study did not use endoscopy to visualize as well as separate these inner membranes. Endoscopy is an accurate modality for both diagnosis as well as therapy.

Conservative treatment was not adapted in our cases with recurrence because this treatment modality is not part of our routine treatment of CSDH. Nevertheless, all our cases with recurrence needed surgery because of the thickened membranes connecting/separating the dura and the thickened arachnoid/pia matters. Studies have shown that, morbidity as well as mortality of CSDH ranges from 0 to 25% and 0–32%, respectively [16, 43, 44]. We observed mortality rate of 1.39%. In all the deceased patients’ treatment of the CSDH was not the direct cause of deaths. Our study is very limited because of the sample size and the number of recurrences. Thus, larger sample sizes will be needed to make definitive conclusions.

## Conclusions

CT scan is capable of detecting of hematoma cavity separations and neomembrane thicknesses. On CT scan, neomembrane thicknesses were seen as abnormal structures relative to normal dura while hematoma cavity separations were seen as two or more subdural cavities. The treatment of patients with CSDH ought to include the identification and resection of abnormal thickened membranes connecting/separating the dura and the thickened arachnoid/pia matters to avoid recurrence. Comparatively, endoscopy showed hematoma cavity separation or neomembrane thickness just as seen during craniotomy.

## Data Availability

The data used in this paper is not publicly available but will be made available on reasonable demand from the corresponding author because of patients’ confidentiality.

## Abbreviations

CI: Confidence interval
CSDH: Chronic subdural hematoma
CT: Computed tomography
HU: Hounsfield unit
OR: Odds ratio
